# Efficacy of a nudge‐based intervention for promoting healthy brain behaviors in people at risk of dementia: A pragmatic cluster‐randomized trial

**DOI:** 10.1002/alz70860_104965

**Published:** 2025-12-23

**Authors:** Jose M Aravena, Hugo Castro, Ronald Poblete, Maria Ines Aravena, Waldo Torres, Paula Vivar, Ester Lara, Marilú Budinich, Patricio Fuentes, Cecilia Albala, Becca R Levy

**Affiliations:** ^1^ Facultad de Medicina Clínica Alemana, Universidad del Desarrollo, Santiago, Region Metropolitana, Chile; ^2^ Department of Social & Behavioral Sciences, School of Public Health, Yale University, New Haven, CT, USA; ^3^ Cultivamente, Santiago, Chile; ^4^ In Medios Agencia Boutique, Santiago, Chile; ^5^ University of Chile, Santiago, Chile; ^6^ Opción Mayor, Santiago, Chile

## Abstract

**Background:**

Although engagement in multiple healthy lifestyles could reduce the risk of dementia, most people refer never have talked about dementia prevention with their healthcare providers. Nudge‐based interventions have demonstrated efficacy in changing real‐life clinical practices. Nonetheless, its effects to modify healthcare providers and patients practices toward dementia prevention has not been tested yet. This was the aim of our study.

**Method:**

A pragmatic cluster‐randomized trial was conducted from April to November 2024 in seven senior centers in Chile. People 60 years or older with cognitive impairment but no dementia were included. Participants in the control centers (n:4) received usual care (physical, social, and cognitive group activities leaded by healthcare providers, two times per‐week) and talks for healthcare providers about dementia’ general aspects. Participants and healthcare providers in the intervention centers (n:3) received the same usual care and talks plus a nudge‐based intervention encouraging AD preventive practices with positive aging images through posters in the centers’ facilities, informative tryptic, and a webpage. Main outcome was a composite healthy brain behaviors score comprising physical, social, dietary, and cardiovascular behaviors. Secondary outcomes were knowledge, healthcare providers practices, and patients’ global cognitive score. Intention‐to‐treat analysis using linear mixed models comparing average treatment effects (ATEs) from baseline over follow‐up were conducted.

**Result:**

A total of 210 participants were included, with mean age 74.8(SD:7.0) and 80.5% women. After 6 months, participants assigned to intervention (n:101) demonstrated larger improvements in the composite healthy brain behaviors score (ATE of SD:0.15, 95%CI:0.02–0.28, p:0.021; a 95.4% improvement compared with control group), refer knowing ways to prevent dementia (intervention‐baseline:32.7%, intervention‐follow‐up: 61.4%, p:0.002; control‐baseline:49.1%, control‐follow‐up:50.9%, p:0.997), a healthcare provider talked about dementia prevention (intervention‐baseline:9.9%, intervention‐follow‐up:21.8%, *p* <0.001; control‐baseline:7.3%, control‐follow‐up:5.5%, p:0.973), and the number of people receiving risk factors management recommendations from healthcare providers (intervention‐baseline:53.5%, intervention‐follow‐up:73.3%, p:0.018; control‐baseline:65.5%, control‐follow‐up:70.9%, p:0.821). No differences in global cognition scores were observed, but the intervention group presented a reduction in cases with scores compatible with moderate cognitive impairment.

**Conclusion:**

Complementing real‐life clinical practice with positive AD prevention nudge‐based information can lead to significative changes in healthy brain behaviors practices from both patients and healthcare providers.

